# Highly enantioselective photo-polymerization enhanced by chiral nanoparticles and in situ photopatterning of chirality

**DOI:** 10.1038/s41467-020-15082-6

**Published:** 2020-03-04

**Authors:** Chenlu He, Zeyu Feng, Sizhen Shan, Mengqiao Wang, Xin Chen, Gang Zou

**Affiliations:** 1 CAS Key Laboratory of Soft Matter Chemistry, Department of Polymer Science and Engineering, University of Science and Technology of China, 230026 Hefei, Anhui China; 2 Department of Chemistry, Boston University, Boston, MA USA

**Keywords:** Conjugated polymers, Nanoparticles

## Abstract

Chiral noble metal nanoparticles has recently gained great interest due to their potential applications including ultrasensitive chiral recognition and asymmetric synthesis. We anticipate that they could be utilized to induce asymmetric photo-polymerization reactions with high enantioselectivity and reactivity. Here, we report such a system. By employing silver nanoparticles modified with cysteine as the chiral inducer, polydiacetylene (PDA) with high chiral asymmetry was obtained from achiral diacetylene monomers triggered with unpolarized UV light. Furthermore, the helical sense of chirality can be controlled by varying the wavelength of UV irradiation. This enables a feasible and economical method to fabricate programmable 2D patterns of chiral PDA with tailored chirality distributions, such as smooth gradients in chirality and micropatterns with tailorable circularly polarized luminescence. Our finding not only opens a pathway for producing programmable chiroptical micropatterns, but also is highly valuable for deeper understanding of symmetry breaking in enantioselective photochemical reactions.

## Abstract (web summary)

Chiral nanoparticles are capable of ultrasensitive detection, characterization, and asymmetric synthesis of chiral organic and biological molecules. Here, the authors use silver nanoparticles modified with cysteine, as the sole chiral source, and unpolarized UV light, to form polydiacetylene with high chiral asymmetry from achiral diacetylene monomers.

## Introduction

Chirality is a ubiquitous and fascinating feature in nature and has been extensively discussed in physics, chemistry, biology, and materials science^1–3^. Chiral conjugated polymers, particularly those with strong circularly polarized luminescence, have recently gained increasing interest as they promise many potential applications in quantum computing, chiral recognition-based biosensing, chiroptical metamaterials, etc.^4^. In the past decades, many strategies have been developed to synthesize chiral conjugated polymers^5^, typically using chiral monomers^6^, dopants, catalysts^7^, or asymmetric external influences including circularly polarized light (CPL)^8,9^, vortex motion^10^, stirring^11^ and other physical forces^12^, as the symmetry breaker.

It would be highly desirable to pattern chiral conjugated polymers in situ at micro/nano-scale with tailorable chiroptical properties as functional photonic elements to be used in chiral optoelectronics and nanophotonics^13^. Traditional lithographic techniques such as electron beam lithography^14–16^ could produce micro- or nano-sized patterns of polymers. However, these techniques are often prohibitively time-consuming and costly to fabricate complicated patterns over a large area, impeding their practical applications. In situ photopatterning of chiroptical polymers would be a more preferred strategy. Ideally, symmetry breaking in photo-polymerization reactions from achiral monomers could be in principle induced by irradiation with CPL^17^. Unfortunately, only small enantiomeric excess can be obtained in typical cases, as evident by their very small asymmetry factors (*g* < 10^−3^)^17–19^. We have recently shown that enantioselectivity of a photo-polymerization reaction would be elevated with superchiral light, i.e. a standing wave formed by two counter-propagating CPL^20^. However, the enhanced optical asymmetry is restricted in very narrow regions near the nodes of the standing wave, making it incompatible with photopatterning^21,22^. Up to date, it remains a great challenge for in situ photopatterning of such polymers with tailorable chiroptical properties in a feasible and economical fashion.

Chiroptical responses can also be enhanced in chiral systems involving noble metal nanoparticles^23^. At least some types of chiral amplifications can be attributed to chiral electromagnetic fields generated by optical excitation of chiral plasmonic nanoparticles^24,25^. Great progresses have recently been made in the fabrication of chiral metal nanoparticles and/or superstructures^26^, and their applications such as enantioselective catalysis have been demonstrated^27–29^. We anticipate that the optical asymmetry of these systems may also promote enantioselectivity as well as reactivity in photo-polymerization reactions. To the best of our knowledge, no such attempts have been reported. Moreover, the preference in handedness is dictated by not only geometry and configuration of the nanostructures, but also the wavelength of optical excitation.

Herein, we demonstrate experimentally that the enantioselective photo-polymerization of achiral diacetylene (DA) monomers can indeed be achieved using cysteine-modified AgNPs, a chiral molecule-NP complex, as the symmetry breaker in the photo-polymerization of PDA. Importantly, the direction of symmetry breaking (the preferred handedness) could be reversed by varying either the chiral configuration of chiral ligands on nanoparticles or the excitation wavelength. This allows us to pattern PDA with a desired helical sense (left- or right-handed) onto a film through photopatterning with unpolarized UV only. Thus, we developed a feasible and economical method to fabricate programmable 2D patterns of chiroptical polymers with chirality and/or chirality distributions. A few examples of patterned PDA films, with tailorable circularly polarized luminescence or capable of enantioselective colorimetric sensing, have been successfully demonstrated.

## Results

### Enantioselective photo-polymerization of PDA

**Fig. 1:**
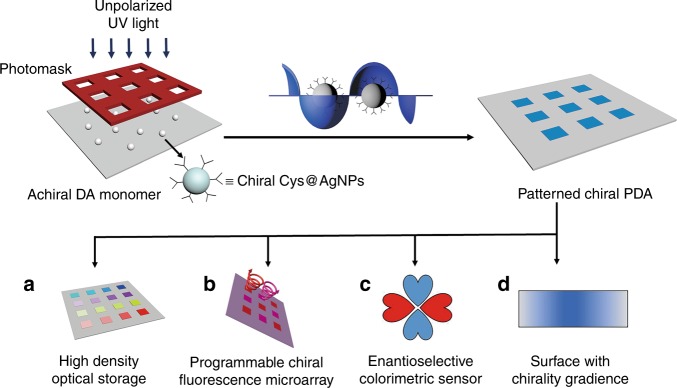
Schematic of photopatterning of chirality in PDA films and their potential applications. Highly asymmetric chiroptical PDA is obtained through enantioselective photo-polymerization assisted with noble metal nanoparticles. Arbitrary 2D patterns and/or distributions of PDA with tailorable chirality can be readily produced by controlling spacial distribution of UV irradiation. Various chiral PDA patterns could be fabricated as designed, such as **a** a microarray of chiral PDA, **b** a display panel with chiral fluorescence, **c** a clover-shaped pattern as an enantioselective sensor, and **d** a gradient of chirality.

Our general strategy to induce highly asymmetric photo-polymerization reactions, as outlined in Fig. 1, utilized chiral noble metal nanoparticles as the symmetry breaker. Initially achiral Ag nanoparticles (~30 nm) were made by tethering either l- or d-cysteine onto surfaces to form the chiral molecule-NP complex (Cys@AgNP), which exhibited moderately strong circular dichroism (CD) signals (Supplementary Fig. 1). In case of d-cysteine, the CD spectrum featured a bisignate pattern with negative signals around 250 nm and positive signals around 290 nm; this is a characteristic manifestation of a positive Cotton effect. If l-cysteine was used instead, the CD spectrum showed a negative Cotton effect due to the mirror symmetry, featuring the same CD pattern but with all signs flipped. Similar CD patterns have been reported by multiple groups and attributed to cysteine ligands chemisorbed on the surface of AgNPs^30^. Interestingly, the wavelength of this CD band coincided with the interband absorption of AgNPs (220−320 nm) rather than their surface plasmon resonance absorption at ~410 nm^31^. Clearly, it is not plasmonic CD. The molecular origin of the strong CD signal has yet been fully understood. Some studies in literature claim it to be directly related to cysteine binding to the metal surface of through a thiolate-silver bond^31^, while others believe that cysteine can dimerize into cystine, which is a large and more polarizable chiral molecule itself^32^.

Importantly, this CD band matches the wavelength of UV light that can readily trigger the polymerization reaction of DA molecules; this enabled us to utilize the chiral Cys@AgNPs as the symmetry breaker in our system. Achiral DA in the vicinity of such chiral Cys@AgNPs should be excited preferably into one of two enantiomers of the excited state (a diradical), probably through a similar mechanism in photo-polymerization directly with CPL^20^. This should eventually lead to a PDA chain with the corresponding handedness: the helical sense of PDA was dictated by both handedness of the ligands and the UV excitation wavelength. Therefore, this allowed the chirality and chirality distribution of PDA films to be rigorously controlled by manipulating the relevant physical parameters (wavelength, intensity, and their spacial distributions) of the UV irradiation. Programmable patterns of PDA including chirality gradients and microarrays with tailorable circularly polarized luminescence could be readily fabricated. As shown in Fig. 1, such patterned films promise a wide range of potential applications, such as high density optical storage (Fig. 1a), where information can be recoded in a four-dimensional space, a programmable array with a 2D distribution of circularly polarized fluorescence (Fig. 1b), which could be used in 3D display or quantum communications, an enantioselective colorimetric sensor (Fig. 1c), which reports chirality of analyte with color changes visible to naked eyes, and gradient chiral surfaces (Fig. 1d), which would be uniquely useful in chiral separations and biomimetics.

**Fig. 2:**
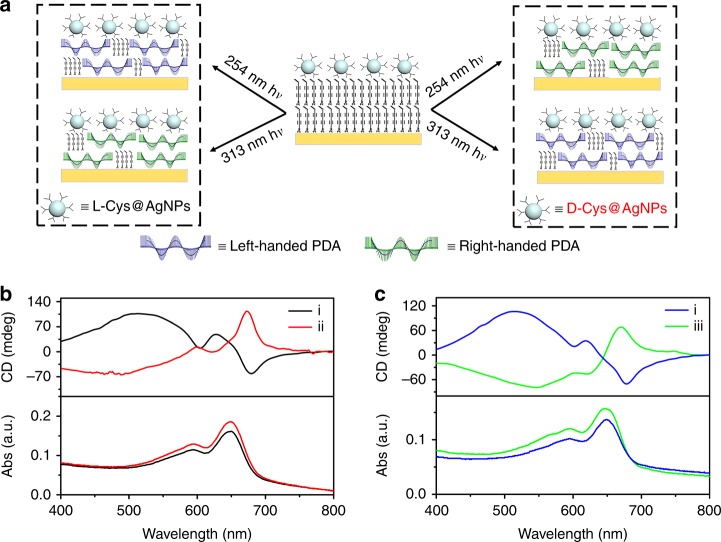
Handedness of PDA depends on both chiral configuration of ligands on plasmonic nanoparticles (L- vs. D-) and wavelength of UV irradiation (254 nm vs. 313 nm). **a** Handedness of PDA obtained with all four possible combinations. Left-handed PDA is obtained using 254 nm irradiation assisted with L-Cys@NPs or 313 nm irradiation assisted with D-Cys@NPs. Right-handed PDA is obtained using 254 nm irradiation assisted with D-Cys@NPs or 313 nm irradiation assisted with L-Cys@NPs. **b** CD spectra showing that chirality of PDA is reversed using (i) l- and (ii) d-Cys@AgNPs upon irradiated with 254 nm unpolarized light. **c** CD spectra showing that chirality of PDA is reversed using (iii) 313 nm irradiation instead of (i) 254 nm unpolarized light in the presence of L-Cys@AgNPs.

As illustrated in Fig. 2, photo-polymerization reactions were carried out by shining unpolarized UV light onto a film of achiral monomers covered with either l- or d-Cys@AgNPs. The samples turned blue with intense absorption maximums at about 643 and 587 nm, confirming the formation of PDA. The characteristic bisignate CD signals of chiral PDA could be observed at this absorption band. PDA obtained using 254 nm UV excitation with l-Cys@AgNPs as the symmetry breaker was left-handed, as proven by the negative Cotton effect with a crossover at 657 nm (black curve in Fig. 2b). If d-Cys@AgNPs were used instead, the corresponding CD spectrum (red curve in Fig. 2b) flipped signs, indicating that the predominant product became right-handed. (To exclude the possible effect of linear dichroism, CD measurements were carried out by rotating the sample about the normal of the film. The variation of CD signals at all rotation angles was small, indicating that the main origin of CD should be the helical chains of PDA, as shown in Supplementary Fig. 2.) The sample polymerized in the presence of either l- or d-Cys@AgNPs was highly asymmetric, with the maximum *g* factors reaching −7.9 × 10^−3^ and 8.5 × 10^−3^, respectively; the *g* factors were of similar magnitude but carried opposite sign, due to the mirror symmetry of these two samples. We note that the enantioselectivity in this system was ~20 times greater than that with direct CPL irradiation without assistance of AgNPs (*g* = −3.7 × 10^−4^ and 4.4 × 10^−4^, respectively, as shown in Supplementary Fig. 3). We believe that this large enhancement effect could be mostly attributed to the enhanced optical asymmetry of Cys@AgNPs.

To further support the conclusion, several control experiments were performed. Firstly, PDA obtained from achiral DA monomers with unpolarized UV radiation showed no chiral preference, as expected (Supplementary Fig. 4a). Secondly, if the AgNPs used were undecorated or decorated with a racemic mixture of cysteine, the resultant PDA under the otherwise same condition showed similar overall absorbance, but no obvious CD signals (Supplementary Fig. 4b, c). Lastly in the case of l- or d-cysteine only (without any AgNPs), very weak CD signals could be detected (Supplementary Fig. 5). (The corresponding CD signals varied strongly with the rotation angle and the net average is very small (Supplementary Fig. 6), indicating that the linear dichroism effect was the main origin of the CD signals.) Clearly, the direct chiral induction effect of cysteine molecules was extremely weak in our system. All these results suggested that the symmetry breaking in the photo-polymerization reactions should be attributed to the enhanced optical asymmetry in the vicinity of Cys@AgNPs, instead of cysteine itself.

Importantly, we found that the preferred helical sense of PDA depended on the excitation wavelength. The CD signals for Cys@AgNPs at 254 and 313 nm carried opposite signs (Supplementary Fig. 1b), indicating that the corresponding nearfields were of opposite handedness. Therefore, by simply varying the irradiation wavelength, one can manipulate the direction of the chiral symmetry breaking in photo-polymerization reactions (Fig. 2). For example, since the CD signal of l-Cys@AgNP at 254 nm was positive (Supplementary Fig. 1b), the local field in resonance with 254 nm radiation should be more left-handed, which would favor the formation of left-handed helical PDA chains (Fig. 2b). To the contrary, the local field generated near the same nanoparticles with 313 nm irradiation was more right-handed (since the CD signal at 313 nm was negative), and the predominant product became right-handed (Fig. 2c). Naturally, the opposite was true for d-Cys@AgNP because of the mirror symmetry. The results of all four possible combinations are tabulated in Supplementary Table 1 for direct comparison. Examining the table allows us to draw the important conclusion: the symmetry breaking was solely determined by the handedness of CD that is in resonance with UV irradiation, rather than the handedness of the chiral ligands tethered on the AgNPs, despite the fact that the latter were the ultimate source of chiral asymmetry in the whole system.

**Fig. 3:**
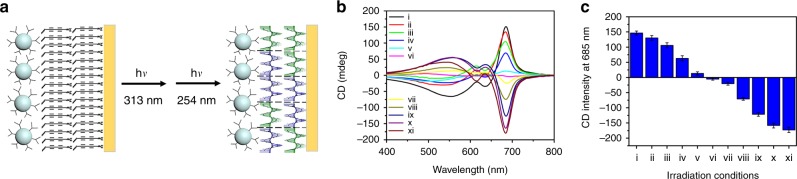
Fine-tuning the degree of chiral asymmetry of PDA with sequential UV irradiation at 313 nm and 254 nm. **a** A mixture of left-handed and right-handed PDA obtained with sequential exposure of UV at two wavelengths. **b** CD spectra and **c** corresponding CD intensities at 685 nm of PDA obtained with different irradiation conditions: (i) 360 s at 313 nm, (ii) 340 s at 313 nm and 10 s at 254 nm, (iii) 320 s at 313 nm and 15 s at 254 nm, (iv) 300 s at 313 nm and 20 s at 254 nm, (v) 280 s at 313 nm and 25 s at 254 nm, (vi) 260 s at 313 nm and 35 s at 254 nm, (vii) 200 s at 313 nm and 40 s at 254 nm, (viii) 140 s at 313 nm and 45 s at 254 nm, (ix) 80 s at 313 nm and 50 s at 254 nm, (x) 20 s at 313 nm and 55 s at 254 nm, (xi) 60 s at 254 nm, respectively. At least four irradiation experiments were performed to obtain the average CD signals, and error bars represented standard deviations of measured CD signals.

Such wavelength dependence allowed us to conveniently control the chiral asymmetry of PDA. The films in Fig. 3 were sequentially irradiated with 254 or 313 nm UV light, each for a given duration of time as listed in the figure caption. This double-exposure process resulted in a mixture of left- and right-handed PDA. The correlation between the measured CD signals of the films and the relative amounts of UV irradiation at two wavelengths were found to be monotonic, as shown in Fig. 3b, c: the CD signal decreased with longer irradiation time at 254 nm and shorter irradiation time at 313 nm, dropping gradually from +146 mdeg at 685 nm (with 313 nm irradiation only) to −174 mdeg at 685 nm (with 254 nm irradiation only). Therefore, the chiral asymmetry of PDA films could be fine-tuned by choosing an appropriate ratio of irradiations with 254 and 313 nm light.

### Patterning of films with enantioselective colorimetric sensing

**Fig. 4:**
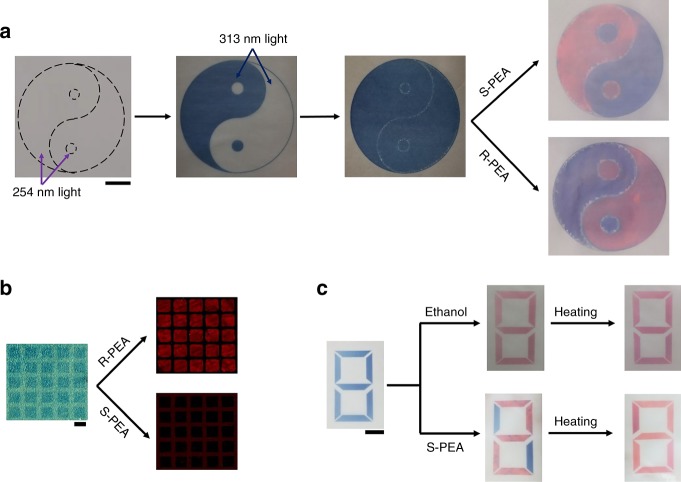
Photopatterning of chiral PDA into various 2D patterns. **a** Tai-Chi patterns where the red and blue regions filled with left-handed and right-handed PDA when stained with a small chiral alcohol. The filling colors are switched for the two enantiomers of the alcohol. Scale bars, 1 cm. **b** A microarray of chiral PDA patches in 5 µm × 5 µm squares and 2 µm spacing. Scale bars, 5 µm. **c** A piece of hidden information coded with chirality that can be deciphered with chiral molecules only. Scale bars, 1 cm.

With chirality tunable by UV irradiation, programmable 2D distributions of chirality in a PDA film could be readily produced through in situ photopatterning using photomasks, following a procedure conceptually similar to patterning of photoresist in standard photolithography. PDA with desired chirality can be selectively polymerized into an arbitrary 2D pattern with UV irradiation passing through a photomask placed directly on top of the film. Herein we elaborate on the procedures to fabricate the Tai-Chi pattern in Fig. 4a as an example. Firstly, achiral DA monomers dissolved in cyclohexane solution was used as an invisible ink and directly printed into a circular region onto a piece of weighing paper using inkjet printing^33^. It was then selectively polymerized in two steps of UV irradiations: in the first step, the Yang regions (including the major area in the up-left and the small circle at the bottom) were irradiated with 254 nm unpolarized light only, then followed with a second step by irradiating the Yin regions (the other half of the circle) with 313 nm. Thus, the obtained PDA was predominantly left-handed in the Yang regions, and right-handed in the other half. Both regions appeared blue with no visual differentiation. To visualize the distinct chirality, we took advantage of the blue-to-red phase transition of PDA which could be triggered by small chiral alcohols such as R- or S-phenylethanol (PEA)^20^. Specifically, S-PEA interacted more strongly with left-handed PDA than right-handed, and accelerated the phase transition of the former (Supplementary Fig. 7). Once the whole piece of paper was wetted with a solution containing S-PEA only, the Yang regions quickly turned red, while the other half remained virtually unchanged (Fig. 4a, last panel). This enantioselective staining revealed the overall pattern as a classic Tai-Chi symbol with red representing Yang and blue representing Yin. The two colors were switched if the other enantiomer of PEA was used. A film with such patterns could be used as an enantioselective sensor, to report handedness of a chiral analyte with color changes visible to naked eyes. In theory, the spacial resolution of this photopatterning technique could reach the diffraction limit of the UV light. Figure 4b presents an array of PDA patches, where the 5 µm × 5 µm squares and 2 µm spacing being selectively polymerized with 313 and 254 nm irradiations, respectively (see Supplementary Fig. 8 for more details).

Various patterns can be designed to suit other circumstances where chiral recognition is necessary or desired. Figure 4c demonstrates another example, in which a message was coded with chirality and therefore decipherable with chiral recognition only. Seven patches of chiral PDA were printed on a piece of paper, together making up a symbol of 8. This symbol was initially blue, and turned into a red 8 if treated with achiral alcohols (or racemic mixtures of alcohols). However, this would cover up the hidden information coded with chirality. Five of the seven patches were actually filled with left-handed PDA, forming a symbol of 2, which could be temporarily revealed by wetting the paper with S-PEA. Heating the whole piece of the paper or simply waiting for several minutes would allow the phase transition of PDA to complete, regardless of its chirality, allowing the coded message to burn after reading automatically.

### Programmable microarray with circularly polarized luminescence

**Fig. 5:**
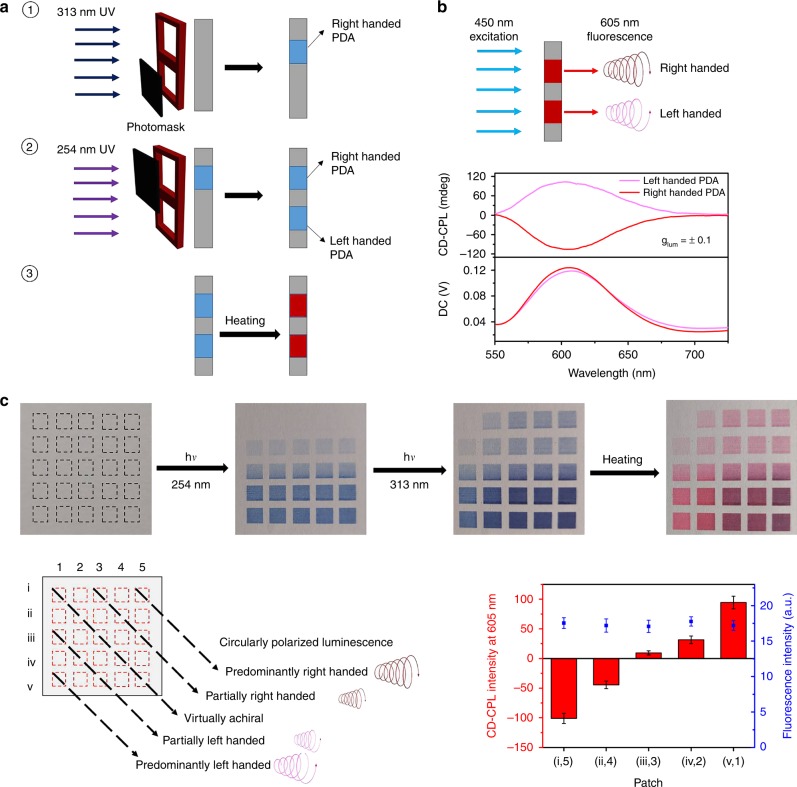
Programmable chiral PDA arrays with circularly polarized fluorescence. **a** Two-step irradiation to pattern right-handed and left-handed PDA sequentially on a film. **b** Either right- or left-circularly polarized fluorescence emitted with high asymmetry factors (*g*_lum_ = ±0.1 at ~605 nm). **c** A matrix of PDA patches with tailorable chirality. The patches in each row are irradiated with varying amounts of 254 nm and in each column with varying amounts of 313 nm, resulting in a 2D matrix of PDA patches each with its own unique combination of chirality and intensity. For example, right-handed PDA is found in the northeast corner, left-handed PDA in the southwest corner, and achiral PDA in the diagonal patches; while chirality varies along the diagonal direction of northeast-southwest, the intensity of fluorescence increases step-wisely along the other diagonal from northwest to southeast. At least three experiments were performed to obtain the average signals, and error bars represented standard deviations of measured signals.

Conveniently, the red phase of PDA emits fluorescence around 605 nm if excited with 450 nm light. Unsurprisingly, the chiral PDA prepared with our method emitted circularly polarized fluorescent light, even if excited with unpolarized 450 nm light. As shown in Fig. 5a, the luminescence asymmetry factor (*g*_lum_) of the left- or right-handed PDA films could reach as high as ±0.1 (Fig. 5b), respectively, which was about ten times higher than that polymerized directly with CPL (Supplementary Fig. 9). Such high optical activity could be attributed to mostly the high asymmetry factor in PDA itself. (Note that the excitation wavelength is not far from the plasmon resonance of AgNPs, and this may influence chiral asymmetry in the fluorescence as well^4^.) The sign and value of *g*_lum_ could also be fine-tuned by simply controlling the ratio between the irradiations with 254 and 313 nm unpolarized light. Using a method similar to that aforementioned, a matrix of square patches of fluorescent PDA was fabricated with each patch featuring a unique chiroptical property of its own, as shown in Fig. 5c. Selective excitation of a certain patch allowed one to read its chirality in fluorescence. Moreover, one can selectively illuminate certain regions to create a desired spacial distribution of circular polarization, which would respond instantaneously to the excitation light (with nanosecond latency due to the nature of fluorescence). Such a device should be useful in fields where information is carried in helical senses of circular polarization, such as 3D display and quantum communications.

### Fabricating a continuous gradient of chirality

**Fig. 6:**
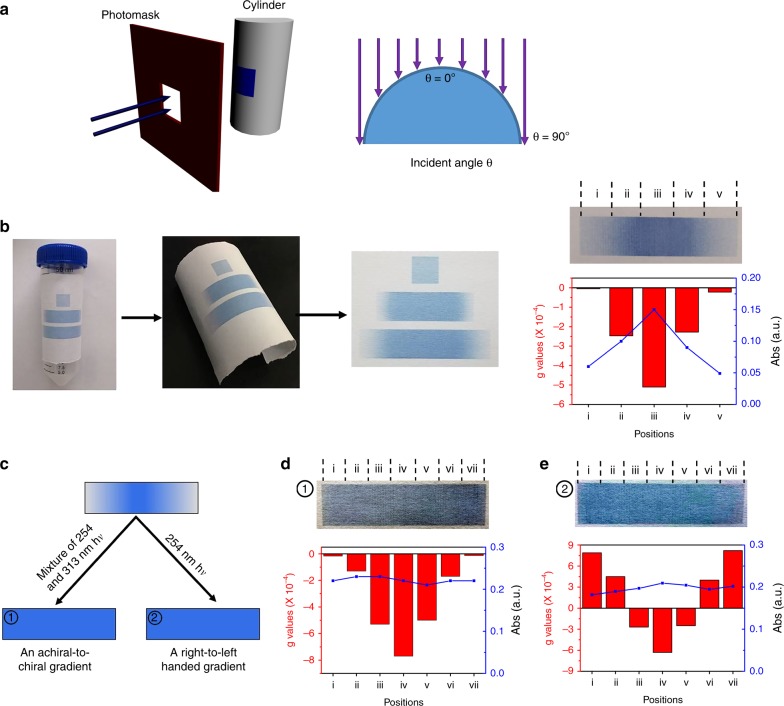
Fabricating a continuous gradient of chirality by taking advantage of surface curvature. **a** Schematic of optical setup in which the incident angle varies with location. **b** Uneven distributions of PDA obtained due to uneven distributions of UV irradiation on the film, when wrapped around a cylindrical object. A rectangular patch of PDA with chirality decreasing gradually from the center to the two edges, forming a smooth gradient, as proven by both its absorption and CD along the lateral locations. **c** Schematic of setup in which a second UV irradiation shining on the sample obtained in (**b**) to generate a seemingly uniform film (same absorption) with varying chirality along the lateral locations. Two different gradients, an achiral-to-chiral gradient in (**d**) and a right-to-left-handed gradient in (**e**) are produced under two different irradiation conditions, ① and ②, respectively.

Our method also allows us to generate chirality gradients and gradient distributions in PDA films. Like chirality itself, chirality gradients are ubiquitous in nature as well, such as in shells of snails^34^_._ A surface with chirality gradient(s) would be uniquely useful in biosensoring, chiral separations, and biomimetics; for example, one could direct the diffusion of a chiral object, such as a biomacromolecule or an even living organism near a surface with a designed gradient of chirality. Such surfaces with chirality gradient would be very difficult to produce with traditional lithographic techniques^35^ but easily accessible using our method. As shown in Fig. 6a, an achiral DA film covered with d-Cys@AgNPs was pre-printed by inkjet printing on a piece of paper. Flexibility of paper allowed it to be rolled into a column around a cylindrical object like a centrifuge tube. A collimated beam of 313 nm UV light passing a square opening illuminated a belt region only on the film. Due to the geometric alignment, the incident angle of the UV irradiation varied naturally from 0° at the center to 90° at the edges, as illustrated. The final film, after being unwrapped and laid on a flat surface, featured a rectangular region with an uneven distribution of PDA horizontally. As proven by the CD spectra shown in Fig. 6b, the chirality of PDA varied with lateral location, with the center being predominantly left-handed and the two edges essentially achiral. In a PDA film produced in this way, a gradient in chirality was necessarily accompanied by a same gradient in absorption. While sometimes this might be a favorable feature, in some other circumstances, a gradient in chirality only while keeping the overall absorption constant could be more preferable, for example, when one desires to single out the effect of chirality. Such a gradient in chirality only could also be readily achieved with our method using a sequential exposure procedure; a film was first prepared with a gradient in the same fashion in the first experiment, then followed with a second step of illumination with a strong and evenly distributed light beam that ensured overexposure of DA to guarantee an even distribution of overall absorption across the sample (Fig. 6c). If the second light beam used was a mixture of 254 and 313 nm that overall generated achiral PDA, we obtained an apparently uniform film except for a chirality gradient, as proven by its absorption and CD spectra at the different lateral locations of the sample shown in Fig. 6d. In a third experiment, the second light beam was chosen to be pure 254 nm instead, which produced PDA with the opposite chirality. Again, a seemingly uniform PDA film was obtained, but in this case containing a hidden gradient in chirality with right-handed PDA at the center and left-handed PDA at the two edges, as shown in Fig. 6e. All the gradients were smooth and continuous without any abrupt changes (Supplementary Fig. 10). The steepness of the gradient could be controlled by varying the curvature and/or curvature distribution of the substrate (e.g., round columns with different diameters, elliptical columns, or more irregular shapes). Even more complicated distribution(s) of PDA could be generated if the film were folded into a 3D structure matching the desired distribution(s) after proper geometric transformations.

## Discussion

We believe that the unusually high enantioselectivity in our system can be largely attributed to a chiral induction effect amplified by the cysteine-modified silver nanoparticles. The ultimate chiral source of our system is the cysteine ligand. Since cysteine (or cystine if they dimerize)^36^ are aligned on the surface of AgNPs and form an organic layer, there should be a cooperative effect in the enantioselective polymerization, since multiple cysteine (and/or cystine) ligands could serve as chiral inducers simultaneously and attribute to asymmetrical growth of a single polymer chain with a certain helicity. In our system, the so-called sergeant-and-soldiers effect^37^ should also be in play, since chiral alignment of one DA molecule should help to align another DA molecule. In addition, we note that DA can be readily polymerized with UV irradiation between 220 and 320 nm; this optical exaction is in resonance with both the CD signals of the AgNPs and their interband absorption^31^. This might induce another chiral amplification effect since the electromagnetic field in the vicinity of AgNPs must be magnified by the absorption, although the enhancement factor at this frequency range is much smaller than at surface plasmon resonance at 412 nm, and the underlining mechanism should be fundamentally different. (See Supplementary Fig. 11 for FDTD simulations of the enhancement factors at 254, 313 and 412 nm). Overall, these effects should collectively contribute to the enantioselectivity and as the result, chiral PDA films with very high asymmetry factors (*g*_lum_ = ± 0.1) can be readily produced and selectively patterned. Interestingly, all of the chiral amplification effects for this photo-polymerization reaction should occur only in the vicinity of Cys@AgNPs, which effectively serves as a chiral nano-reactor.

To make chiroptical materials truly useful in practice, even larger chiral asymmetry (greater *g* values) is preferred or even necessary. It is now well established that CD coupled with plasmonic resonance depends on the size of the nanostructures as well as their constitution, geometry, and the relative locations of neighboring particles^38,39^. The CD of our system (the frequency of which coincides with interband transitions) has been found to vary with these parameters as well, but in different ways. This opens a possibility of further improvement of our system by systematically studying the detailed mechanism of the enhancement effect, to obtain PDA with even greater chiral asymmetry. For example, we could optimize the metal NPs to generate more surface atoms on a particular crystal facet, or to produce more hot spots^24^ by controlling aggregation of these NPs. This would impart even greater chiral amplification in the photo-polymerization reactions and/or other types of photochemical reactions. An alternative strategy could be adopted is to combine the current system with the superchiral light setup^21^ to take advantages of the amplification in both near- and far-fields.

Photopatterning chiroptical polymers with very high asymmetry factors would enable many applications. During the process of enantioselective photo-polymerization in our system, the wavelength information of UV is effectively translated into and recorded with chirality of the polymerized PDA. While at the same time, photopatterning with UV allows the spacial distribution and the intensity of UV to be preserved with high fidelity in the locations and the concentration of PDA in a thin film. Therefore, this system could potentially be used as high density optical storage. The four parameters (two dimensions in space, one in concentration, and one in chirality) are mutually orthogonal, forming a 4D information space, with each parameter independently controllable. The four parameters recorded in every PDA patch can be read by its optical properties, in either absorbance or fluorescence, as we have demonstrated earlier.

In summary, we discover that highly asymmetric chiroptical polymers (*g*_lum_ = ± 0.1) can be produced from achiral monomers through asymmetric photo-polymerization with unpolarized UV light, by employing chiral noble metal nanoparticles as symmetry breaker. The helicity of polymers can be controlled by varying the wavelength of UV. Furthermore, we utilize this system to establish a feasible and economical protocol to fabricate programmable 2D patterns of chiroptical polymer films with tailored chirality, which could be potentially useful in chiral optoelectronics and photonics devices.

## Methods

### Materials

All chemicals used were of analytical grade or of the highest purity available to us. Silver nitrate (AgNO_3_, 99%), amino acids, cyclopentanone, ethylene glycol were obtained from Sigma-Aldrich (St. Louis, MO, USA) and used as received. 10,12-Pentacosadiynoic acid (DA) was purchased from Tokyo Chemical Industry Co., Ltd., and purified by dissolving in cyclopentanone and subsequently filtrating to remove the polymer before using. All glassware was thoroughly cleaned with freshly prepared 3:1 HCl/HNO_3_ (aqua regia) and rinsed thoroughly with water prior to use. Cys@AgNPs (Supplementary Fig. 1a) were synthesized by decorating bare, achiral silver NPs (30 nm in diameter) with optically pure (l- or d-) cysteine molecules, following a literature procedure with minor modifications^40^.

### Characterizations

All UV−Vis spectra were recorded on a Shimadzu UV-2550 PC spectrophotometer. CD spectra were measured by using a JASCO CD spectrometer J-810. Transmission electron microscopy images were recorded on a JEOL-2000 microscope. Dynamic light scattering was recorded on NanoBrook Omni. Atomic force microscope (AFM) was recorded on AFM SPA-300HA. Circular polarized luminescence was measured using a JACSO CPL-300 spectrometer.

### Enantioselective photo-polymerization

In a typical experiment, a 200 μL solution containing l-or d-Cys@AgNPs was drop-casted on a DA film spin-coated on a quartz substrate (1.5 cm × 1.5 cm). The films were kept in darkness for 12 h before being irradiated with UV light. The intensities of unpolarized UV light at 254 or 313 nm were set to be 3 and 2 mW cm^−2^, respectively. Enantioselective photo-polymerization of the DA films can by triggered by irradiation with unpolarized UV light. The sense of chirality of the resultant PDA is dictated by the chirality of the plasmonic particles, as well as wavelength of the UV light, with the details being elaborated in the main text.

## Supplementary information

Supplementary Information

## Data Availability

The authors declare that all data supporting the findings of this study are available within this article and Supplementary Information files, and also are available from the authors upon reasonable request.
