# Supplementary material for: Highly enantioselective photo-polymerization enhanced by chiral nanoparticles and in situ photopatterning of chirality
Source: Nat Commun. 2020 Mar 4;11:1188. doi: 10.1038/s41467-020-15082-6 (PMC7055214; doi:10.1038/s41467-020-15082-6)
Supplement: Supplementary file 1 — Supplementary Information [file 41467_2020_15082_MOESM1_ESM.pdf]

Supplementary Information

Highly Enantioselective Photo-polymerization Enhanced by Chiral Nanoparticles and in situ Photopatterning of Chirality

He et al.

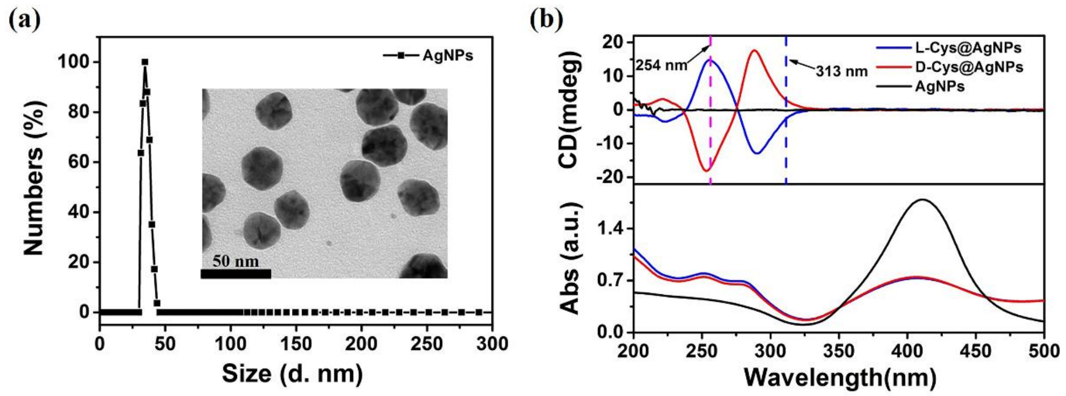

Supplementary Figure 1| DLS data and CD spectra. (a) DLS size of AgNPs, inset image is the TEM images of AgNPs. **(b)** The CD and UV-Vis spectra of AgNPs, L- and D-Cys@AgNPs.

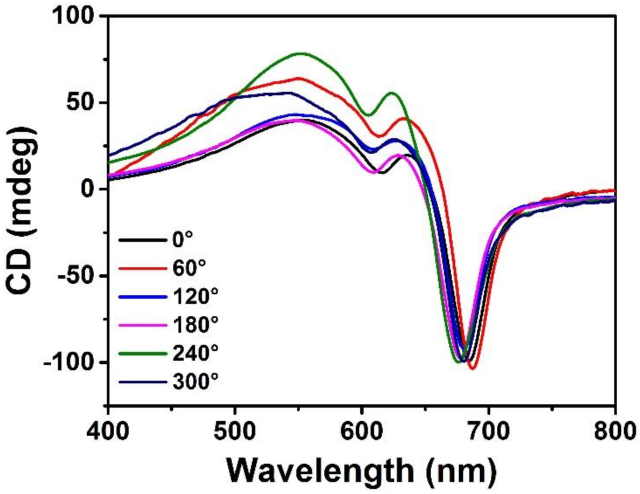

Supplementary Figure 2| CD spectra. The CD spectra at various rotation angles about surface normal for the samples polymerized with 254 nm unpolarized light assisted with L-Cys@AgNPs. The signal intensity hardly changed with various rotation angle, indicating that the main origin of CD signals should be the helix formation of PDA chains.

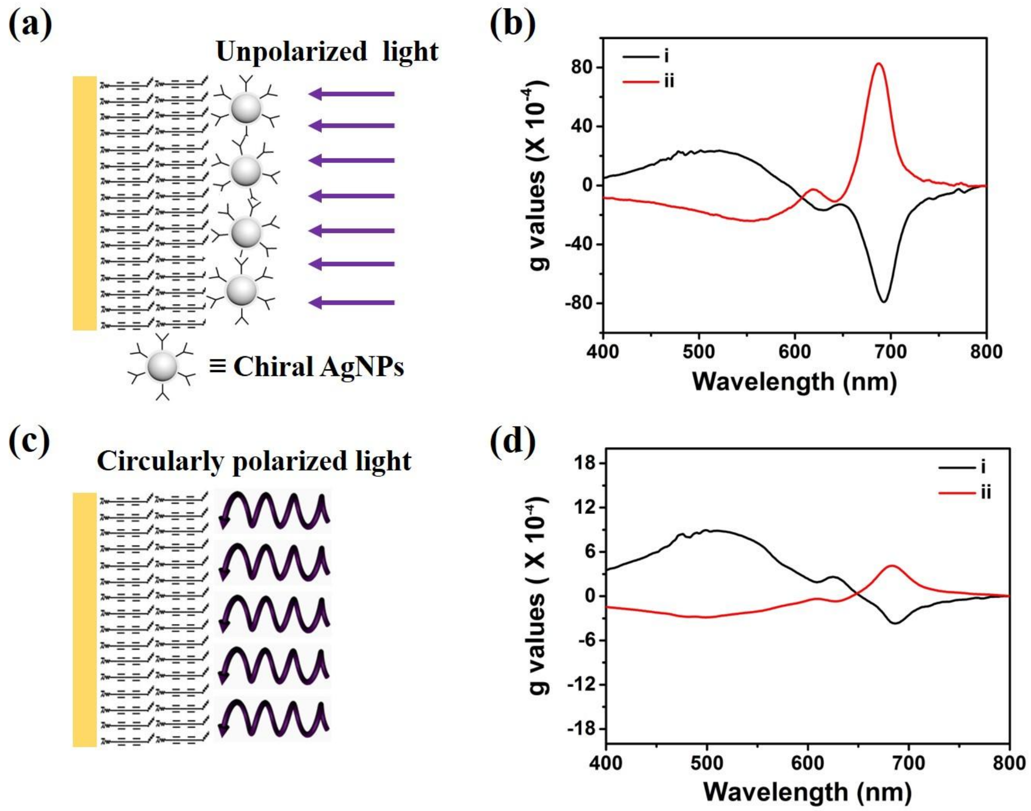

Supplementary Figure 3| Anisotropy spectra. **(a)** Schematic of chiral PDA films upon irradiation with 254 nm unpolarized light assisted with chiral AgNPs. **(b)** The g values for chiral PDA films upon irradiation with 254 nm unpolarized light assisted with (i) L- or (ii) D-Cys@AgNPs, respectively. **(c)** Schematic of chiral PDA films upon irradiation with CPL. **(d)** The g values for chiral PDA films upon irradiation with: (i) left- or (ii) right-handed CPL, respectively.

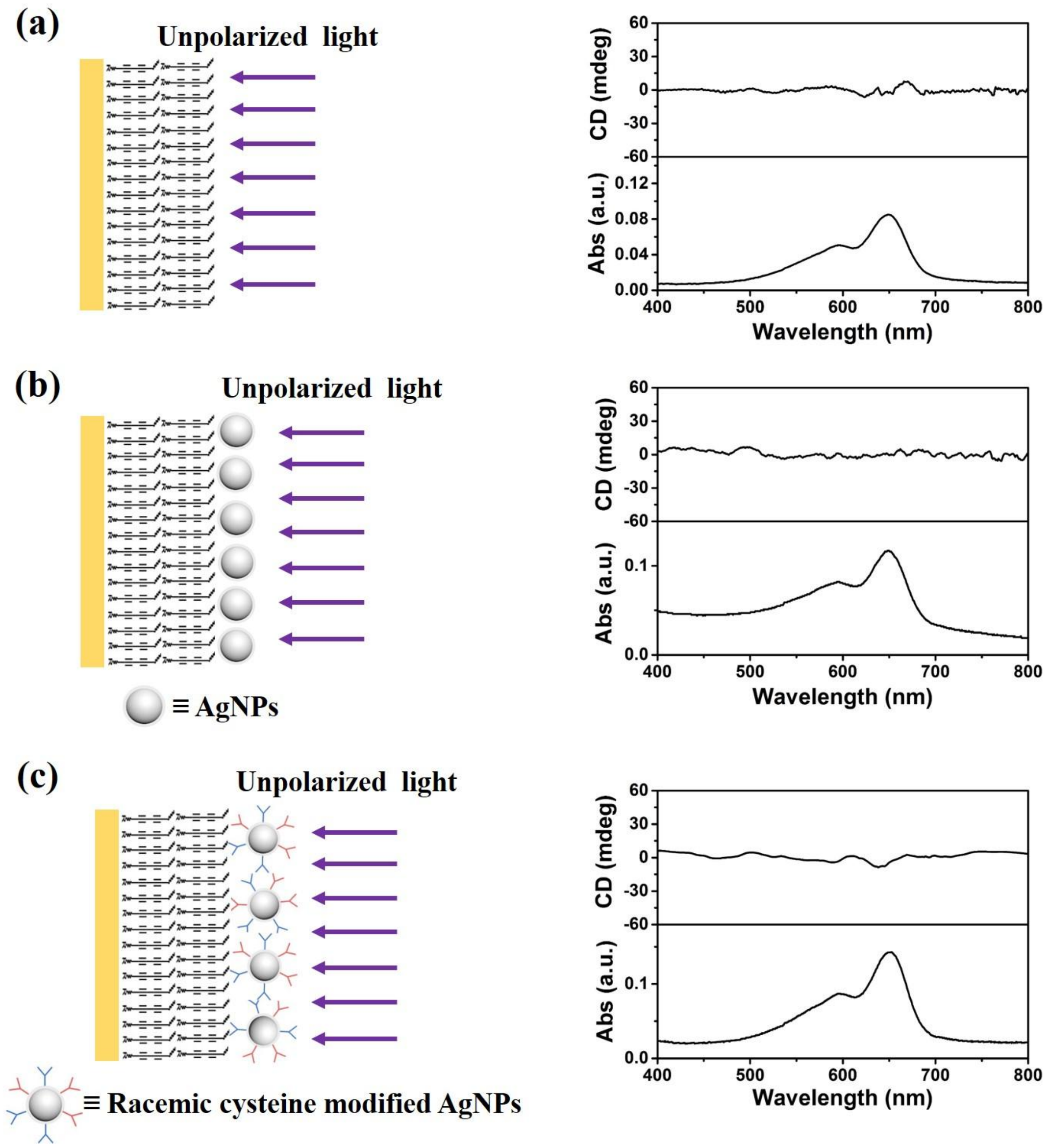

Supplementary Figure 4 | The control experiments. (a) Schematic illustration, averaged CD and UV-vis spectra of PDA films after irradiated with 254 nm unpolarized light. **(b)** Schematic illustration, averaged CD and UV-vis spectra of PDA films upon irradiation with 254 nm unpolarized light assisted with undecorated AgNPs. **(c)** Schematic illustration, averaged CD and UV-vis spectra of PDA films upon irradiation with 254 nm unpolarized light assisted with AgNPs decorated with a racemic mixture of cysteine.

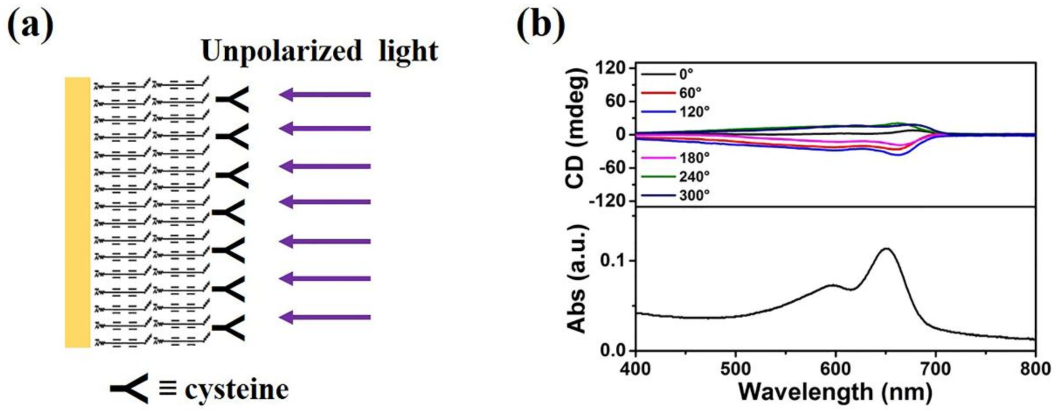

Supplementary Figure 5| Another control experiment (a) Schematic of PDA films covered with optically pure cysteine molecules (5×10^{-4} M) upon irradiation with 254 nm unpolarized light. **(b)** CD spectra at various rotation angles about surface normal for the samples polymerized with 254 nm unpolarized UV light in the presence of optically pure cysteine molecules drop-casted on the film. The signal intensity changed with various rotation angle, indicating that the main origin of this asymmetry should be at least partially attributed to linear dichroism effect.

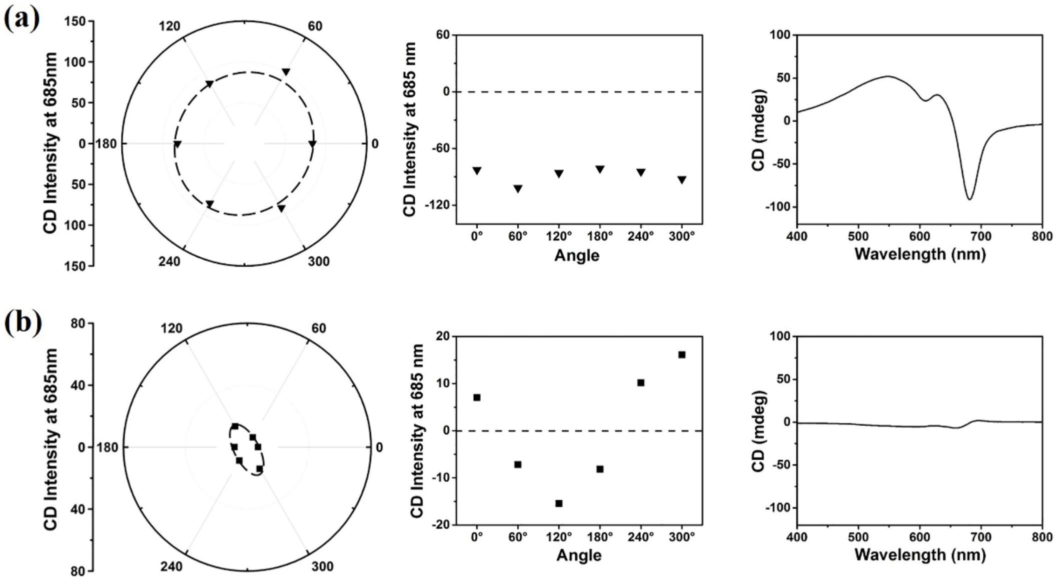

Supplementary Figure 6| Comparing the Angular dependence of CD of PDA polymerized (a) with Cys@AgNP and **(b)** with cysteine molecules only as the symmetry breaker. The CD data presented in Fig. S2 and Fig. S5 were re-plotted in two different types of graphs, clearing showing the angular dependency. In case of Cys@AgNP, the signal is always negative, while in case of cysteine only, the CD signals can be either positive or negative, and vary greatly depending on the angle. The arithmetic averages were calculated and plotted as well. Obviously the averaged CD signals with Cys@AgNP are much greater than those obtained with cysteine molecules only, indicating that the former is a much better chiral inducer in our system.

Supplementary Table 1| Comparing the CD signal of Cys@AgNPs with the handedness of resultant PDA at different wavelength.

	L-Cys@AgNPs		D-Cys@AgNPs	
**CD sign at**	254 nm	313 nm	254 nm	313 nm
	**positive**	**negative**	**negative**	**positive**
**Irradiation condition**	254 nm	313 nm	254 nm	313 nm
**Optical activity of PDA**	Left-handed	Right-handed	Right-handed	Left-handed

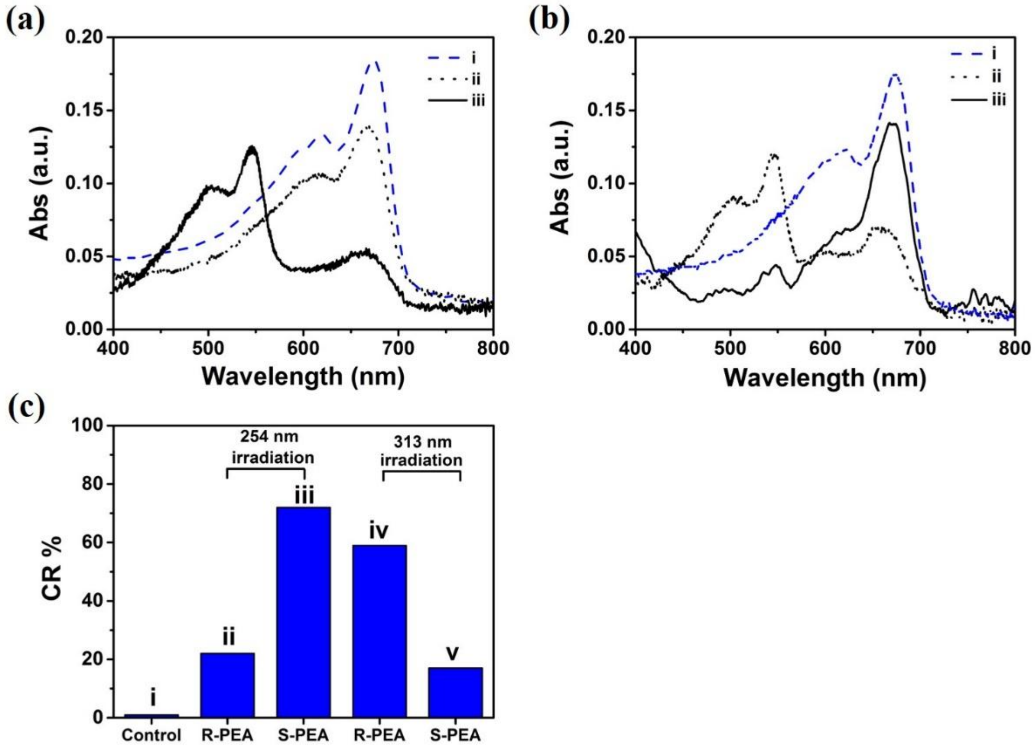

Supplementary Figure 7| UV–vis spectra and CR values. UV–vis absorption spectra of chiral PDA films irradiated with **(a)** 254 nm or **(b)** 313 nm unpolarized light assisted with L-Cys@AgNPs: (i) before treatment, or upon wetting into 0.03 M (ii) R- or (iii) S- PEA solution, respectively. **(c)** The CR values of chiral PDA films prepared with 254 nm and 313 nm unpolarized light assisted with L-Cys@AgNPs on response to R- or S- PEA solution. The colorimetric response (CR) is defined as the relative change in percent of blue phase PDA, defined as $CR = [PB_0 - PB_1]/PB_0 \times 100\%$, where PB_0, PB_1 was the initial and final percent of blue phase in PDA films before and after immersion into PPA enantiomers solution, respectively.

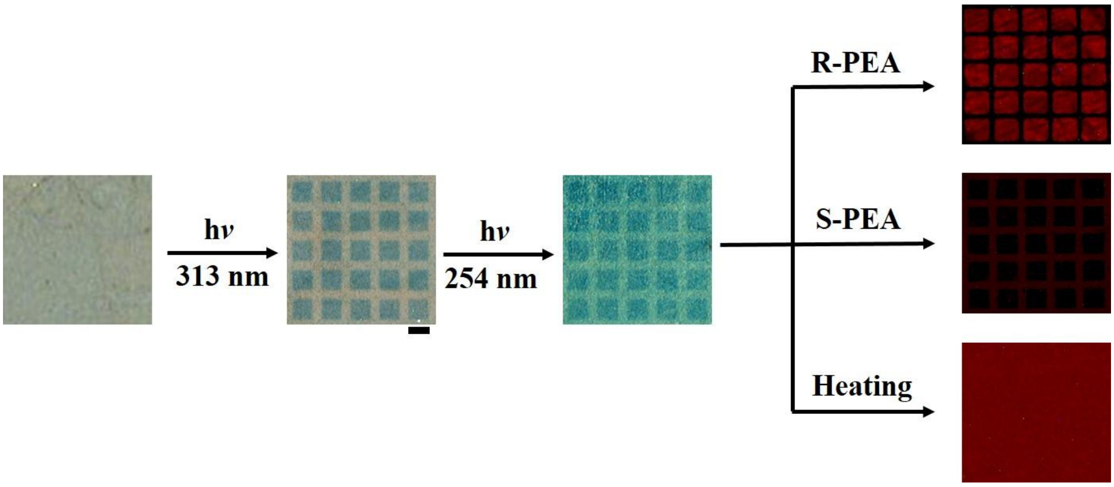

Supplementary Figure 8| Fabrication of PDA micropattern. Microscopy image of fabricating PDA micro-patterns using a modified two-step exposure technique and fluorescent microscopy image of chiral PDA films with micro-pattern upon treated with R-, S-PEA or heating, respectively. The L-Cys@AgNPs were used in this experiment. Scale bars, 5 μm.

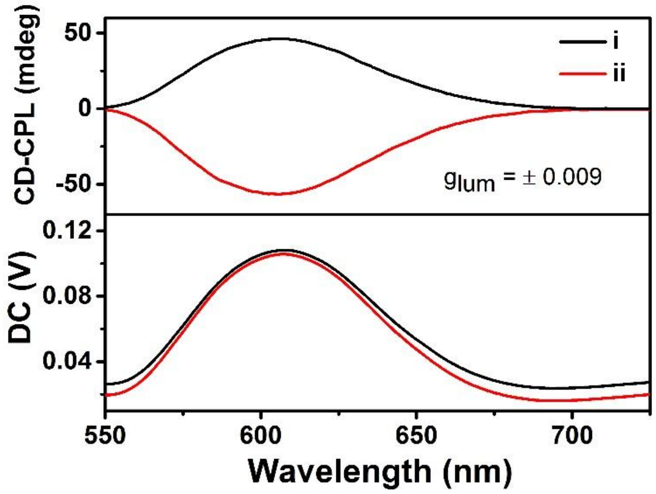

Supplementary Figure 9| CPL spectra. The circular polarization fluorescence spectra and corresponding luminescence dissymmetry factor (g_{lum}) of chiral PDA films irradiated with (i) left-handed or (ii) right- handed CPL.

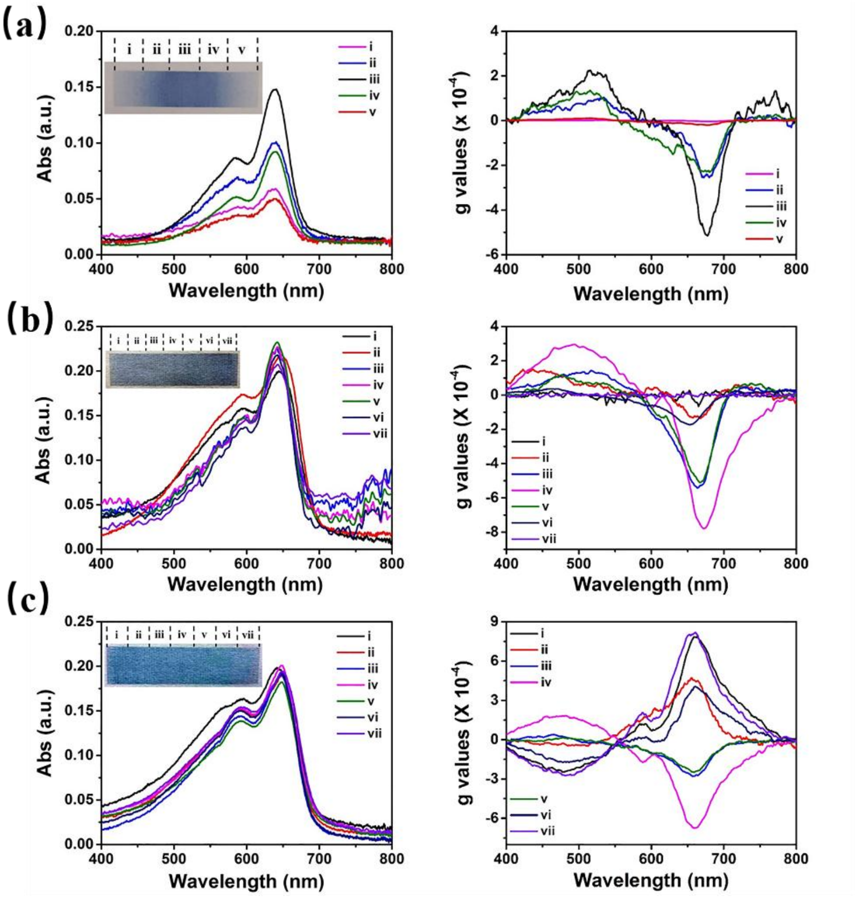

Supplementary Figure 10| UV-Vis adsorption and anisotropy spectra. UV-Vis spectra and g values for PDA films at different lateral locations in the three samples shown in (a) Fig. 6b, (b) Fig. 6d and (c) Fig. 6e in the manuscript, respectively.

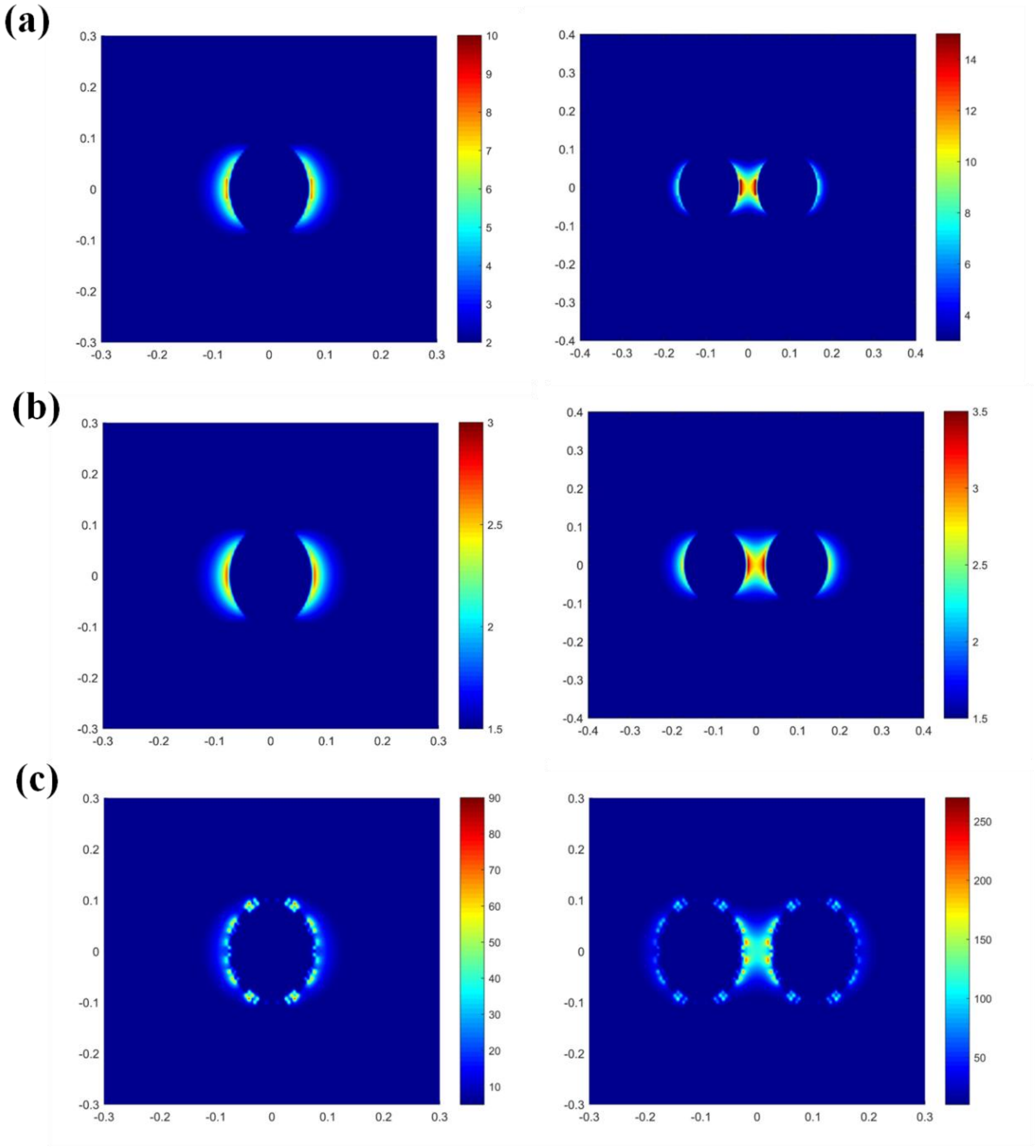

Supplementary Figure 11| FDTD simulations. Simulations of the enhancement factor of AgNPs (~30 nm) upon irradiated with (a) 254 nm, (b) 313 nm and (c) 412 nm unpolarized light.
